# Nurturing care practices for children with developmental disabilities in sub-Saharan Africa: A scoping review protocol

**DOI:** 10.1371/journal.pone.0291839

**Published:** 2024-05-06

**Authors:** Silas Onyango, Margaret Nampijja, Paul Otwate, Nelson Langat, Linda Oloo, Kenneth Okelo, Patricia Kitsao-Wekulo

**Affiliations:** 1 Early Childhood Development (ECD) Unit, Human Development Theme, African Population and Health Research Center, Nairobi, Kenya; 2 Department of Psychology, School of Philosophy, Psychology and Language Sciences, University of Edinburgh, Edinburgh, United Kingdom; Ahmed Physiotherapy and Research Center, BANGLADESH

## Abstract

**Background:**

The majority of children with neurodevelopmental disorders **(**NDDs) reside in low- and middle-income countries (LMICs). NDDs are a public health concern in countries in sub-Saharan Africa (SSA). Nurturing care has been recommended as a pathway for addressing the developmental needs and unlocking the full potential of children, including those with NDDs. However, little information exists on the strategies to support children with NDDs using the Nurturing Care Framework in many countries in SSA. This review aims to synthesize information on nurturing care practices for children with NDDs in SSA. The review will also determine gaps in the provision of nurturing care for children with NDDs. Further, the review will highlight the drivers of care as well as the experiences of the caregivers.

**Methods:**

The review will be implemented in six steps: specification of the research question, identification of relevant studies, selection of studies to be included, extracting, mapping, and charting the data, collating, summarizing, and reporting the results, and stakeholder consultation. We propose a database search followed by a manual search for the literature synthesis. We will search the following electronic databases: PubMed, ScienceDirect, Scopus, Open Grey and African Journals Online (AJOL). All studies published after May 2018 to May 2023 that include relevant terms will be identified and included. The research team will develop a data extraction form for use in capturing relevant information from each of the included studies. A patterning chart that will summarize and analyze the key findings of each article will be created.

**Discussion:**

We anticipate that the study will provide evidence on the existing nurturing care practices and unearth gaps in the provision of nurturing care for children with NDDs. Key determinants of care and the experiences of the parents/caregivers of children will also be identified. The study will provide key recommendations on interventions to improve the quality of care for children with NDDs. Through this study, awareness of the unmet nurturing care needs of these children will be increased. The evidence generated may assist policymakers and stakeholders in addressing the needs of children with NDDs.

## Background

Globally, it is estimated that over 53 million children currently live with neurodevelopmental disorders (NDDs), and the majority of these children live in low- and middle-income countries (LMICs) [1]. Children with NDDs face deficits in neurological and brain functioning that considerably impair their cognition, communication, mobility, and/or social interaction. NDDs, or childhood disabilities, are a significant cause of poor development in children and continue to be a public health concern in many countries in SSA [2]. Nurturing care has been recommended as a pathway for addressing the developmental needs and unlocking the full potential of children, including those with NDDs [3]. *Nurturing care* refers to a stable caregiving environment that provides for good health, adequate nutrition, opportunities for early learning, responsive caregiving, and safety and security [4]. Empirically, all children need nurturing care to thrive; however, children with NDDs need more intensive nurturing care strategies not only to survive but also to thrive, as they are at a higher risk of poor developmental outcomes.

With nurturing care, known and modifiable risk factors for developmental disorders, such as poor maternal health-seeking behavior and family violence, can be monitored and effectively mitigated. Children with NDDs are often at risk for maltreatment, violence, and neglect due to unfavorable cultural beliefs about disability, discrimination, and family and community stigma [2]. Limited resources in most settings in SSA, where most of these children live, further constrain their care as the demands for their needs are high. Moreover, persistent parental stress and negative feelings associated with having a child with NDDs present a challenge [1]. Studies on young children in LMICs have generally excluded those with NDDs, and therefore little is known about the importance of interventions such as nurturing care to support child growth and development.

The World Health Organization, UNICEF and the World Bank Group in collaboration with the Partnership for Maternal, Newborn & Child Health and the Early Childhood Development Action Network launched the Nurturing Care Framework to help children survive and thrive in order to achieve their full potential [4, 5]. Since its launch, the Framework has been implemented across the globe, with countries in SSA being the focus. However, the nurturing care of children with NDDs who require more support has not received adequate attention. Further, little information exists on the strategies of using the Framework to support children with NDDs in many countries in SSA. The main objective of the proposed scoping review is therefore to identify, explore, and map the literature on the nurturing care practices for children with NDDs in SSA. The review will also highlight the existing gaps in the provision of nurturing care for children and determine the drivers of the quality of nurturing care provided. Further, the review will highlight the experiences of caregivers of children with NDDs in relation to the care provided. It is anticipated that the evidence generated from this study will inform appropriate interventions or practices that can inform policy on nurturing care for children with NDDs across the SSA region. This will ensure that children living with disabilities in low-resource settings receive the nurturing care they need to thrive and achieve their full potential.

The research questions are:

1. What are the current nurturing care practices for children with NDDs in SSA?
2. What gaps exist in the provision of nurturing care for children with NDDs?
3. What are the key determinants/drivers of the quality of nurturing care provided to children with NDDs?
4. What are the experiences of parents/caregivers of children with NDDs in relation to the nurturing care provided?

## Methodology

Using a scoping review approach, we seek to answer the overarching question: "What are the current nurturing care practices for children with NDDs in SSA?” We chose a scoping review method because it will provide a broader perspective on the existing nurturing care practices across different age ranges. The review will be based on the five components of the nurturing care [4] and will follow the proposed methods by Arksey & O’Malley [6] and advanced by Levac and colleagues [7]. The scoping review will be implemented in six stages namely, i) specification of the research question, ii) identification of relevant studies, iii) study selection, iv) data extraction, mapping and charting, v) collating, summarizing and reporting the results and vi) stakeholder consultation [8]. We will follow the preferred reporting items for systematic reviews and meta-analyses extension for scoping reviews (PRISMA-ScR) checklist [9] to select studies. The team used Microsoft Excel to manage the review process. The PICO framework will guide the title and abstract screening (**Table 1**).

**Table 1:** PICO framework for determining eligibility of review questions.

Criteria	Determinants
**Population**	Caregivers and their children with disabilities (intellectual disabilities, autism spectrum disorders, cerebral palsy, attention deficit and hyperactive disorders (ADHD), and sensory disorders)
**Intervention**	Nurturing care for early childhood development (good health, adequate nutrition, responsive caregiving, opportunities for early learning, and safety and security)
**Comparison**	Children without disabilities
**Outcomes**	Access to quality and effective nurturing care services
**Study setting**	Sub-Saharan Africa

## Identifying relevant studies

The research team will search published and unpublished literature from all the key relevant electronic databases **(Table 2)**. All studies published after May 2018 to date will be searched using search terms including “developmental disabilities,” “nutrition,” “health,” “nurturing care,” “responsive care,” “early learning,” “safety,” “security,” “children with disability,” “ADHD,” “autism,” “cerebral palsy,” “learning disorder,” “intellectual disorder,” “communication disorder,” and “sub-Saharan Africa.”. In addition, we will search websites such as the World Health Organization (WHO) to identify any potential literature or publications relevant to our study’s main question. Relevant unpublished literature will be identified through a search of conference abstracts, organizational reports, and ProQuest Dissertation & Theses Global. If the search produces too many irrelevant studies, we will consider revising the search strategy to include only relevant terms that could yield reasonable results. The Framework was launched in May 2018, thus the choice of the period of the scoping review.

**Table 2:** A draft search strategy from PubMed.

	Date of search	Search engine used	Number of publications retrieved
(("children with disabilities "[MeSH Terms] OR ("developmental disabilities"[All Fields] AND "health"[All Fields]) OR "nutrition" [(("children with disabilities "[MeSH Terms] OR ("developmental disabilities"[All Fields] AND "health"[MeSH Terms]) OR "nutrition"[MeSH Terms] OR "early learning"[All Fields]) OR ("safety and security"[MeSH Terms] OR "Responsive care"[All Fields] OR "early learning"[All Fields]) OR ("safety and security"[MeSH Terms] OR "Responsive care"[All Fields] AND “sub-Saharan Africa"[All Fields] AND ("2018/05/01"[PDAT]: "2023/05/31"[PDAT]))	17-07-2023	PubMed	2706

## Database searching

We will employ database and manual search to complete the synthesis. Most studies have used database searches to complete scoping reviews, while manual searches are less common. We propose a combination of the two search methods. The review team will carry out the reviews independently.

## Systematic manual search

The five components of the nurturing care make the topic under review interdisciplinary in nature, and therefore some relevant articles may not be found in databases or in other journals that may not be in databases. Therefore, we will supplement the database search with a manual search to access as many articles as possible. Based on the recommendations by Teare and Taks [10], a systematic manual search will be conducted in three steps. The first step will be to select top journals based on the five components of nurturing care and their relation to NDDs. The journals will be identified by the impact factor above one. The research team will then search for articles published after May 2018 that are related to the research topic by keywords. The articles will be selected based on their titles and abstracts, as well as a full-text reading. The articles that meet the criteria will eventually be included in the selection. Finally, after selecting the relevant articles in the top journals, the lists will then be checked to identify other relevant journals, followed by a manual search of journals related to the study objectives. Steps 2 and 3 will be repeated until we reach saturation.

## Systematic database search

**Fig 1:**
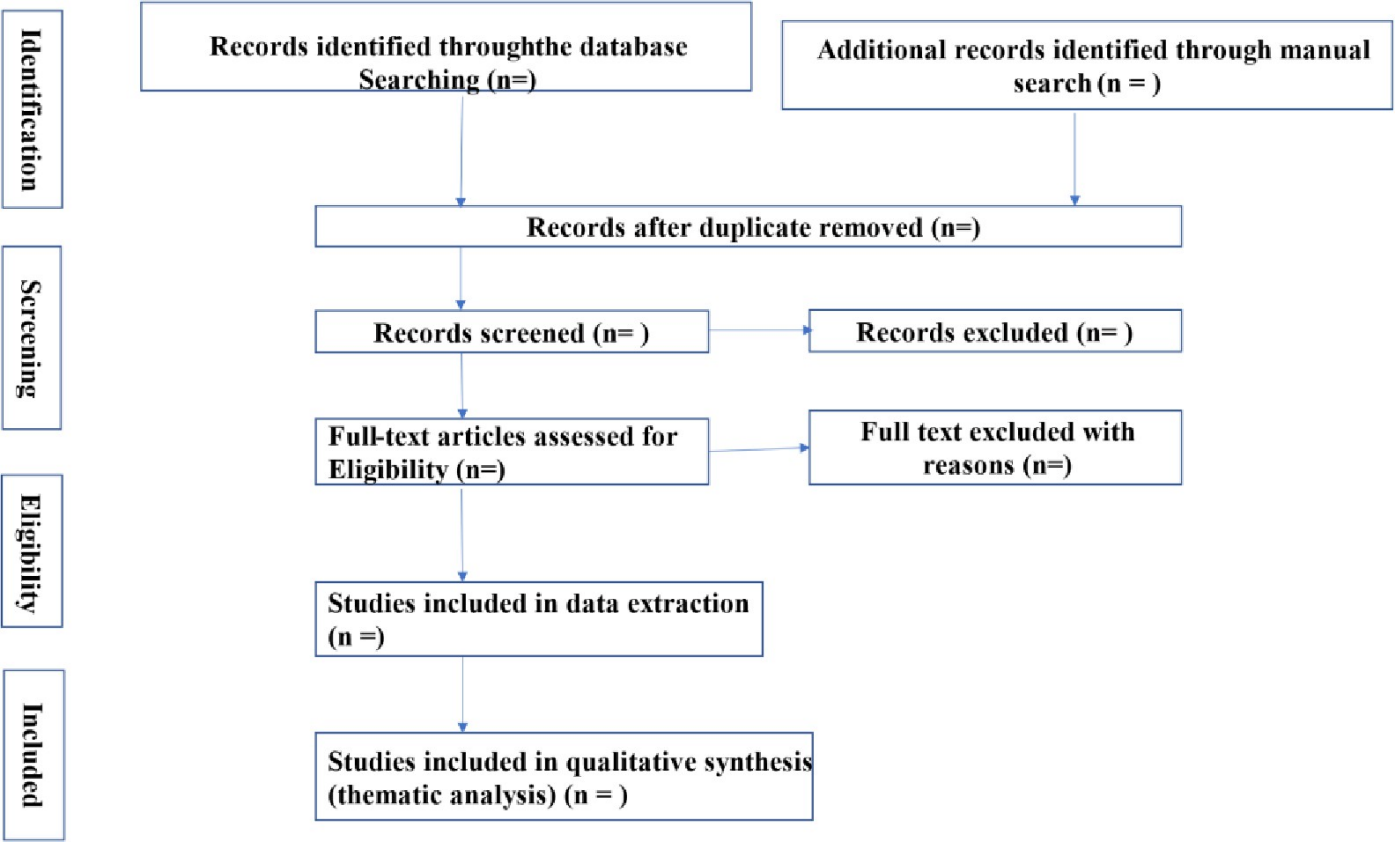
Study selection procedure.

The electronic databases to be searched are PubMed, ScienceDirect, Scopus, Open Grey, and African Journals Online (AJOL). The systematic data search will follow two levels of screening: i) a title and abstract review, and ii) a full-text review. At the first level of review, each of the authors will independently screen the abstracts and titles against a set of inclusion criteria. In the second level, the researchers will independently assess the full-text articles that made it through the screening stage to ascertain that they meet the inclusion criteria. Full-text articles will be reviewed a second time by a third reviewer, and any further disagreements about eligibility at the full-text review level will be resolved through discussion until consensus is reached (**Fig 1**).

### Inclusion and exclusion criteria

The studies that will be included must meet the following criteria: i) they must focus on children ages 0–5 years with NDDs; ii) they must report any of the five components of the nurturing care (health, nutrition, opportunities for early learning, responsive caregiving, and safety and security); iii) they must be published after May 2018, iv) they must include participants from SSA; and v) they must be published in the English language. Any article that will be found relevant by the reviewers and is in line with the research questions will be included in the full review. Studies that report participants outside SSA, have been published before 2018 and are not published in the English language will not be included.

### Charting the data

To capture data electronically, the research team will develop a data extraction form that will capture relevant information from each of the included studies. The form will include information on study identification (using the last name of the first author and year of publication), title, aim, setting, population, sampling method, data collection method, outcomes, data analysis, conclusion, or most relevant findings. The data extraction form will also have a section for comments by the authors (**Table 3**). The form will be reviewed by all investigators before implementation to ensure that it accurately captures important information.

**Table 3:** Data extraction form.

Author
Date of publication
Study title
Study aim/objective
Type of study design
Study setting
Study population
Sampling method
Data collection methods
Data analysis method
Significant findings
Conclusion

## Collating, summarizing, and reporting the outcomes

The Patterns, Advances, Gaps, Evidence of Practice, and Research Recommendations (PAGER) framework will be used to analyze and report the outcomes [11, 12]. After completion of article searches, an analysis of the findings will be undertaken. The first step of the analysis will be to create a patterning chart that will summarize and analyze the key findings of each included article, deductively bringing them together under unique key themes based on the five components of the nurturing care. We will then describe the progression of these patterns (themes), reflecting the dynamic state of the use of nurturing care practices for children with NDDs in SSA. The team will then identify research gaps in the articles, especially in the provision of nurturing care for children with NDDs. In addition, we will take note of areas that have been extensively explored and do not require further exploration. After identifying the gaps, we will extract from each article information useful to practitioners, academics, and policymakers. The information provided will be based on the informed understanding of the authors of this review. Finally, from the identified gaps and information, we will provide practical recommendations to key stakeholders on the best practices and priority areas to support children with NDDs. These recommendations will also be useful in the design of future research on this topic.

## Discussion

The review aims to provide evidence on existing nurturing care practices for children with NDDs in sub-Saharan African countries. The review also aims to identify key determinants of care provided to children with NDDs as well as the experiences of the parents/caregivers of children with NDDs. Further, the review will highlight existing gaps in the provision of nurturing care for children. The review will provide key recommendations on practical best practices and interventions for improving the quality of care and developmental outcomes for children with NDDs. This review will also contribute to increased awareness of the nurturing needs of children with NDDs in SSA. Policymakers and stakeholders may use the evidence generated to address existing implementation gaps for this vulnerable population.

The evidence generated will be limited to sub-Saharan African Countries and may suffer from publication bias, which is why we chose a scoping review approach. The review is also limited to the English language, which may bias the evidence. In addition, the review only considers the common NDDs; (ADHD, cerebral palsy, autism spectrum disorders, learning disorders, communication disorders, and intellectual disorders), excluding all other disabilities, further biasing the evidence.

## Supporting information

S1 Checklist PRISMA-P (Preferred Reporting Items for Systematic review and Meta-Analysis Protocols) 2015 checklist: Recommended items to address in a systematic review protocol*. (DOC)
